# Preparation of Graphite Oxide Containing Different Oxygen-Containing Functional Groups and the Study of Ammonia Gas Sensitivity

**DOI:** 10.3390/s18113745

**Published:** 2018-11-02

**Authors:** Liming Luo, Tongjiang Peng, Mingliang Yuan, Hongjuan Sun, Shichan Dai, Long Wang

**Affiliations:** 1 School of Mineral Processing and Bioengineering, Central South University, Changsha, Hunan 410083, China; luoliming@csu.edu.cn (L.L.); shicandai@csu.edu.cn (S.D.); wanglong6344@163.com (L.W.); 2 Institute of Mineral Materials & Application, Southwest University of Science and Technology, Mianyang, Sichuan 621010, China; pengtongjiang@swust.edu.cn (T.P.); hongjuansun@yeah.net (H.S.)

**Keywords:** reaction temperature, hydrophilic, oxygen-containing functional groups, chemical bonding, ammonia gas sensitivity

## Abstract

A series of graphite oxide samples were prepared using the modified Hummers method. Flake graphite was used as the raw material and the reaction temperature of the aqueous solution was changed (0 °C, 30 °C, 50 °C, 60 °C, 70 °C, 80 °C, and 100 °C). X-ray diffraction, Fourier-transform infrared spectroscopy, Raman spectral analysis, X-ray photoelectron spectroscopy, and contact angle tests were performed to characterize the structure, chemical bonding, type, and content of oxygen-containing functional groups of the graphite oxide samples. The results showed that the type and content of each oxygen-containing functional group could be controlled by changing the reaction temperature with the addition of water. As the temperature of the system increased, the degree of oxidation of the graphite oxide samples first increased and then decreased. Too high a temperature (100 °C) of the system led to the formation of epoxy groups by the decomposition of some hydroxyl groups in the samples, causing the reduction of oxygen-containing functional groups between the graphite layers, poor hydrophilic properties, and low moisture content. When the system temperature was 50 °C, the interlayer spacing of the graphite oxide samples was at its highest, the graphite was completely oxidized (C/O = 1.85), and the oxygen-containing functional groups were mainly composed of hydroxyl groups (accounting for approximately 28.88% of the total oxygen-containing functional groups). The high content of hydroxyl and carboxyl groups had good hydrophilic ability and showed the highest moisture content. The sample at 50 °C had better sensitivity to ammonia because of its high hydroxyl group and carboxyl group content, with the sample showing an excellent profile when the ammonia concentration was 20–60 ppm.

## 1. Introduction

Graphene is a novel two-dimensional carbon material that has attracted a great deal of attention in recent years due to its unique electronic structure [1,2,3] and excellent optoelectronic properties [4,5,6,7]. Graphite oxide [8] is an intermediate product of graphene oxide, which is obtained by the Hummers method, and has a lot of special properties [9], such as a large specific surface area [10], extremely high ion-exchange property [11], and abundant oxygen-containing functional groups on the surface [12]. Differences in oxygen-containing functional groups are not only reflected in the physical and chemical properties (e.g., hydrophilic ability [13] and polar molecular adsorption [14,15]) of graphite oxide, but they also affect the integrity of the graphene structure [16,17] and the distribution of surface defects [18]. Based on different reaction conditions, the structure, morphology, defect distribution, and chemical bond of graphene products have been studied, which is of great significance not only for the controlled and accurate preparation of graphene, but also for further development and utilization of graphene.

Graphite oxide, also known as graphite acid, is a compound with an indefinite quantity of carbon, hydrogen, and oxygen elements. Previous studies on graphite oxide have mainly focused on its formation process [19,20], structure [21,22], and evolution of surface chemical groups [23]. For example, studies have found that HNO_3_ and H_2_SO_4_ intercalated into graphite and formed graphite intercalated compounds [24], which is a necessary process to prepare graphite oxide. Studies looking at ways to improve the Staudenmaier method for preparing graphite oxide discovered the (C_2_F) n-type graphite structure model [20,21] via the fluorinated graphite method. Furthermore, researchers have studied the evolution of various groups in graphite oxide with different oxidation degrees by means of the solid nuclear magnetic field [12,25,26] to more accurately analyze the structure of graphite oxide and the type of chemical groups on its surface. These studies found that there might be groups such as carbonyl groups [27], carboxyl groups [28], ether groups [23,29], and peroxy groups [12] on the surface of the graphite oxide, and at least two magnetically inequivalent sites of hydroxyl groups [30]. Moreover, these two types of hydroxyl groups can bond at any time to form epoxy groups, where the hydroxyl group forms a deformed tetrahedron with four carbon atoms on the six-membered ring of the carbon plane, causing the flat carbon oxide plane to warp [31,32]. Simultaneously, researchers have studied the physical and chemical properties of graphite oxide and found that the surface of graphite oxide is hydrophilic [33,34] owing to its large number of polar groups, the large number of hydroxyl groups gives the oxide an excellent ion-exchange performance [35,36], and different oxygen-containing groups of graphite oxides show excellent gas sensitivity to specific gases [37,38,39]. Based on these characteristics of oxygen-containing functional groups on the surface of graphite oxide, researchers have prepared composite materials with excellent properties using graphite oxide or by combining graphite oxide with other materials, such as graphite oxide photocatalysts [40], polybenzimidazole/graphite oxide composite electrodes [41], chitosan/graphite oxide composite adsorbents [42], and polyaniline/graphite oxide conductive composites [43]. At present, graphite oxide research is more focused on how to make use of these properties, but there has been no in-depth study of the precise and controlled preparation of graphite oxide. In particular, the type and content of oxygen-functional groups have scarcely been reported to date.

In the previous studies of our research group on the preparation [44,45,46], structure, and physicochemical properties of graphite oxide have found that the amount of KMnO_4_ and concentrated H_2_SO_4_ and the reaction time during the middle temperature stage have a significant effect on the oxidation degree of graphite oxide. While the reaction time during the low temperature stage oxidizes the graphite oxide product, the degree of influence is small. Previous research by the author Luo found that temperature changes during the high temperature and water addition stages had a great influence on the degree of oxidation of the graphite oxide and the type of functional groups. To further investigate the influence of water temperature conditions on the structural characteristics and surface functional groups of graphite oxide, the modified Hummers method was used to prepare a series of graphite oxide samples with different oxygen functional groups by controlling the reaction temperature during the water addition stage. The present study then looked at the changes in structure, chemical bonding, content, and type of oxygen-containing functional groups to achieve accurate and controllable content and type of oxygen-containing functional groups of graphite oxide.

## 2. Experimental

### 2.1. Raw Materials and Reagents

Raw materials (natural flake graphite, carbon 90–99.9%, 75 µm) were purchased from Qingdao Shenshu Graphite Products Co., Ltd. (Qingdao, China). Analytical grade potassium permanganate (KMnO_4_), hydrogen peroxide (H_2_O_2_), concentrated sulfuric acid (H_2_SO_4_), and hydrochloric acid (HCl) were purchased from Chengdu Kelong Chemical Factory (Chengdu, China). The experimental water was ultrapure water with a resistivity of >18.25 MΩ·cm.

### 2.2. Sample Preparation

Graphite oxide was prepared by the modified Hummers method, wherein the mass ratio of graphite to KMnO_4_ was 1:4. The experiment consisted of three stages: (1) Low temperature stage: 160 mL of concentrated H_2_SO_4_ was added to a 500 mL dry beaker and stirred in an ice water bath (0 °C). Slowly, 4.0 g of graphite powder was added and stirred for 20 min to fully disperse, then 16.0 g of KMnO_4_ powder was slowly added, and stirring continued at 0 °C for 180 min; (2) Middle temperature stage: after the low temperature stage, the temperature of the water bath was controlled at 37 °C for 60 min; (3) High temperature stage: after the middle temperature stage, 300 mL of ultrapure water was added at room temperature (25 °C), and the temperature of the control system was altered to 0 °C, 30 °C, 50 °C, 60 °C, 70 °C, 80 °C, and 100 °C, with an ice water bath used for the 0 °C treatment. After the addition of water was completed, an appropriate amount of 5% H_2_O_2_ was added until no bubbles formed in the system, and then a quantitative amount of 5% HCl solution was added. The graphite oxide solution stood for 12 h, after which the supernatant liquid was discarded, and 5000 mL of ultrapure water was added. After several repetitions, the graphite oxide solution was washed until the pH showed neutrality, and there was no SO_4_^2−^ in the filtrate (detected by BaCl_2_ solution). The gel was filtered and placed in an oven at 60 °C for 12 h to obtain a yellow-brown graphite oxide sample. Based on the different reaction temperatures, the samples were labeled GO-X, where X = 0, 30, 50, 60, 70, 80, and 100.

### 2.3. Characterization

An X’pert MPD Pro-type X-ray diffractometer (PANalytical, Almelo, The Netherlands) was used for X-ray diffraction (XRD). X-ray photoelectron spectroscopy (XPS) used an XSAM 800 multi-function surface analysis electron spectrometer (Kratos, Manchester, UK). Fourier-transform infrared spectroscopy was performed on a Nicolet-5700 infrared absorption spectrometer (Nicolet, Madison, WI, USA). Raman spectral analysis used an InVia laser Raman spectrometer (Renishaw, London, UK).

The gas sensing performance was analyzed using a WAS-30A gas sensing test system (Zhengzhou Yusheng Instrument Co, Ltd., Zhengzhou, China). Instrument parameters were as follows: acquisition speed 1 time/s, test voltage was 5 V, and system integrated error was less than ±1%. The test process was as follows: a certain concentration of ammonia was first injected into the closed box to compel the ammonia concentration in the closed space to reach the test concentration, the resistance of the sample gradually decreased, and the ammonia sensitivity of the sample was analyzed via the resistance change. After the resistance stabilized, the closed box was opened and the ammonia gas was removed. The recovery time of the sample was tested by the change in resistance. The above process was repeated for different ammonia concentration tests, adding different concentrations of ammonia gas (20 ppm, 40 ppm, and 60 ppm) in a confined space. To eliminate the interference of humidity on the components, the principle of stable relative humidity on the saturated salt solution was used to maintain the humidity above the saturated NaCl solution (humidity 75.3%) and then tested.

## 3. Results and Discussion

### 3.1. Color and Dispersion

**Figure 1:**
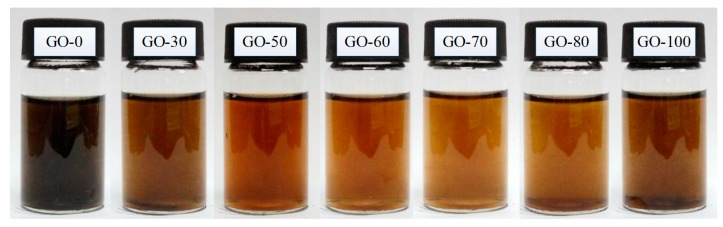
Color change of the graphite oxide dispersion prepared at different reaction temperatures.

The color changes in the graphite oxide dispersion prepared at different reaction temperatures are shown in Figure 1. As the reaction temperature increased, the color of the graphite oxide dispersion gradually changed from brownish black to pale brown and pale brownish-yellow, and finally to dark brown.

When the reaction temperature was 0 °C, the graphite was not completely oxidized owing to the lower reaction temperature and the sample dispersion was poor. Therefore, the dispersion exhibited a mixed color (brownish black) of graphite and graphite oxide. As the reaction temperature increased, the graphite was gradually completely oxidized, and the color of the graphite oxide dispersion gradually changed from brownish black to pale brown (30 °C and 50 °C) and pale brownish-yellow (60 °C and 70 °C) because the dispersibility of graphite oxide was enhanced, and thus the light transmittance increased. When the reaction temperature increased to 80 °C, some of the hydroxyl groups joined to form epoxy groups, the content of carboxyl groups slowly increased, and the increase in the content of epoxy groups and carboxyl groups caused the sample to darken and appear brownish-yellow. Lastly, when the reaction temperature reached 100 °C, due to the high temperature hydrothermal action, the content of hydroxyl groups was greatly reduced, epoxy groups was slowly increased, and the oxidation degree of graphite was lowered, resulting in a decrease in the light transmittance of the sample and the color changing to dark brown.

### 3.2. Contact Angle

**Figure 2:**
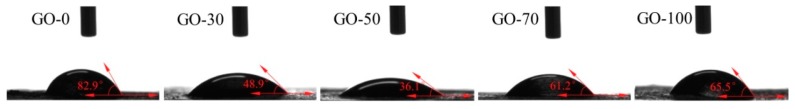
Contact angle of graphite oxide samples prepared at different reaction temperatures.

Results from the hydrophilic tests of the graphite oxide samples prepared at different reaction temperatures are shown in Figure 2. As the reaction temperature increased, the contact angle of the graphite oxide sample decreased from 82.9° (GO-0) to 36.1° (GO-50), and then gradually increased to 65.5° (GO-100), indicating that the reaction temperature had a great influence on the hydrophilic ability of the graphite oxide samples. When the reaction temperature was 0 °C, the graphite was not completely oxidized, being mainly composed of hydroxyl groups, epoxy groups, and a small quantity of carboxyl groups. Although hydroxyl groups and carboxyl groups have a strong affinity with water [47], the hydrophilic ability of the sample was low owing to the low total content of these groups mentioned above, and the water contact angle was poor. When the reaction temperature was 30 °C, 50 °C, and 60 °C, the graphite was completely oxidized, the surface contained a large quantity of hydroxyl groups and carboxyl groups, and the water affinity of hydroxyl groups and carboxyl groups was good; therefore the sample had good hydrophilic ability and a low contact angle. When the reaction temperature increased to 70 °C, 80 °C, and 100 °C, the content of carboxyl groups in the graphite oxide only increased slightly. However, a large amount of hydroxyl groups joined to form epoxy groups, and the water affinity of epoxy groups was poor; therefore, the hydrophilic ability of the sample deteriorated, and the contact angle increased.

### 3.3. Moisture Content and Liquid-Solid Ratio

**Figure 3:**
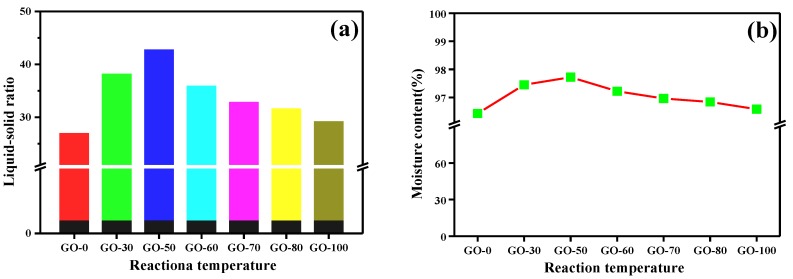
Liquid-solid ratio and moisture content of graphite oxide samples prepared at different reaction temperatures: (**a**) Liquid-solid ratio, (**b**) Moisture content.

To further illustrate the effect of reaction temperature on the oxygen-containing functional groups of graphite oxide, we tested the moisture content (Figure 3a) and calculated the liquid-solid ratio of the graphite oxide samples (Figure 3b). The moisture content of the graphite oxide samples prepared at different reaction temperatures was above 96%, and as the reaction temperature increased, the moisture content first increased and then decreased. The liquid-solid ratio was first 27:1 (GO-0) and increased to 43:1 (GO-50), and then gradually decreased to 29:1 (GO-100), wherein the liquid-solid ratio of the GO-50 sample was 1.5 times higher than that of the GO-0 sample. The hydrogen bonding ability of carboxyl groups and a water molecule is the strongest, hydroxyl groups is the second strongest, and epoxy groups is the weakest. When the reaction temperature was 0 °C, and because the graphite was not completely oxidized at this time, although hydroxyl groups was dominant, the total hydroxyl groups and carboxyl groups content was low, and a large amount of epoxy groups was present, resulting in poor hydrophilic ability of the sample, which was manifested in a low moisture content. With increasing reaction temperature, the graphite was gradually oxidized completely, and a large amount of hydroxyl groups and a small amount of carboxyl groups existed in the edge and surface of the graphite. The GO-50 sample had a good hydrophilic ability and the highest moisture content. When the reaction temperature continued to increase to 100 °C, the degree of oxidation of the graphite further increased, and hydroxyl groups in the structure was initially removed and joined to form epoxy groups. Although the carboxyl groups content increased, the content increase ratio was lower than the removal rate of hydroxyl groups. And to the extent that, the presence of a large amount of epoxy groups resulted in a decrease in the spacing of the graphite oxide layers while also lowering the hydrophilic ability and moisture content.

### 3.4. Structural Characterization

**Figure 4:**
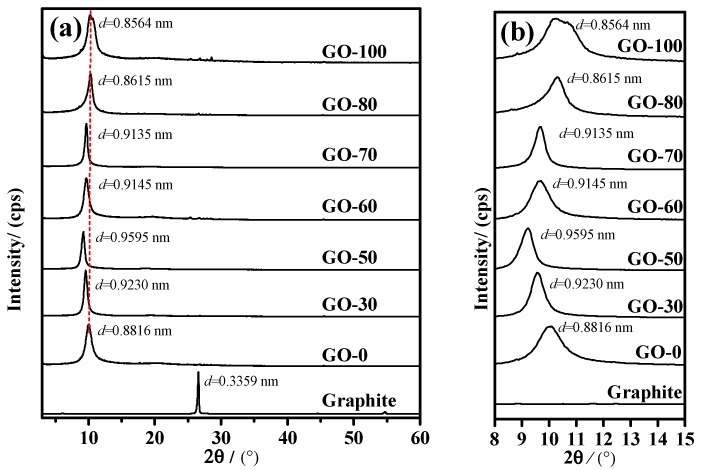
XRD patterns of graphite oxide samples prepared at different reaction temperatures: (**a**) XRD pattern between 3–60°, (**b**) XRD diffraction pattern between 8–15°.

The XRD patterns of the graphite oxide samples prepared at different temperatures after adding water during the high temperature stage are shown in Figure 4. In Figure 4a,b, all samples had strong and sharp diffraction peaks at 2θ = 10° for the different temperatures, which corresponded to the characteristic diffraction peak of the maximum bottom surface spacing of the graphite oxide. The presence of the surface characteristic diffraction peak (d_001_) indicates that all graphite samples were partial or complete oxidized to graphite oxide at the temperatures mentioned above. When the temperature of the water addition was 0 °C, the characteristic diffraction peak of the maximum ground spacing of the graphite oxide (d_001_ = 0.8816 nm) appeared in the GO-0 sample at 2θ = 10.02° and the comparative graphite sample (d_001_ = 0.3359 nm) showed a significant increase. However, the d_001_ value did not reach the sample with a higher degree of oxidation (d_001_ = 0.9 nm) [20], because under low temperature conditions, the oxidation degree of the graphite oxide was low, and the content of oxygen-containing functional groups on the surface and the interlayer was also low. With an increase in the temperature of the system after the addition of water, the characteristic diffraction peak of the maximum bottom surface spacing of graphite oxide shifted to a low angle, d_001_ value rapidly increased to 0.9230 nm (GO-30) and 0.9595 nm (GO-50), and the graphite in the sample was almost completely oxidized. The graphite oxide had the most oxygen-containing functional groups and was mainly composed of hydroxyl groups (approximately 28.77–30.01% of the total amount of the oxygen-containing functional groups).

With a further increase in temperature, the characteristic diffraction peak of the maximum bottom surface spacing of graphite oxide shifted to a higher angle, and d_001_ value was reduced to 0.9145 nm (GO-60) and 0.9135 nm (GO-70). During this time, the oxygen-containing functional groups in the graphite oxide were rich, hydroxyl groups was still dominant (approximately 25.27–27.92% of the total oxygen-containing functional groups), and the content of carboxyl groups and epoxy groups gradually increased. When the temperature was increased to 80 °C and above, the d_001_ value of the graphite oxide was rapidly reduced to 0.8615 nm (GO-80) and 0.8564 nm (GO-100), and the oxygen contents of the sample surface and the interlayer were reduced. Simultaneously, some of the hydroxyl groups in the sample joined to form epoxy groups, resulting in a decrease in the maximum bottom surface spacing of the graphite oxide. This indicated that an increase in the reaction temperature significantly changed the interlayer structure of the graphite oxide.

**Figure 5:**
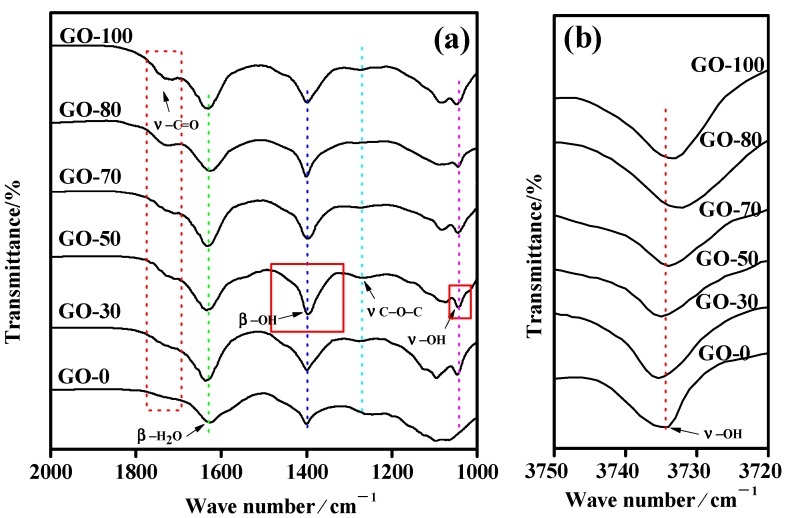
Infrared spectra of graphite oxide samples prepared at different reaction temperatures: (**a**) Infrared spectra between 2000–1000 cm^−1^, (**b**) Infrared spectra between 3750–3720 cm^−1^.

The Fourier-transform infrared spectroscopy spectra of the graphite oxide samples prepared at different reaction temperatures are shown in Figure 5a,b. All the graphite oxide samples had absorption peaks at 3730–3740 cm^−1^ (Figure 5b), 1735 cm^−1^, 1631 cm^−1^, 1400 cm^−1^, 1262 cm^−1^, and 1046 cm^−1^, indicating that the graphite oxide contained various oxygen-containing functional groups. Among these were the C−OH stretching vibration peak at 3730–3740 cm^−1^, the −C=O stretching vibration peak at 1735 cm^−1^, and the bending vibration peak of the graphite oxide surface and the interlayer water molecules at 1631 cm^−1^. At 1400 cm^−1^, the −OH bending vibration peak occurred; at 1262 cm^−1^, the C−O−C stretching vibration peak of graphite oxide was seen; and at 1046 cm^−1^, the C−OH stretching vibration peak was present. With an increase in reaction temperature, the absorption peak intensity at 1735 cm^−1^ increased gradually, and the absorption peak intensity at 1631 cm^−1^ and 1400 cm^−1^ increased first and then decreased, whereas the intensity of the absorption peak at 3730–3740 cm^−1^, 1262 cm^−1^ and 1046 cm^−1^ scarcely changed.

The intensity of the absorption peaks at 1735 cm^−1^ gradually increased owing to the increase in the reaction temperature, and the content of carboxyl groups in the graphite oxide samples continuously increased. However, the intensity of the absorption peak at 3730–3740 cm^−1^ and 1046 cm^−1^ scarcely changed, indicating that the graphite oxide sample contained hydroxyl groups, but because of the influence of the adsorbed water, the change in the functional group content was not reflected in the infrared absorption peak intensity. The absorption peak intensity at 1631 cm^−1^ and 1400 cm^−1^ first increased and then decreased, indicating that, as the reaction temperature increased, the moisture content of graphite oxide increased first and then decreased. Although a large amount of adsorbed water was removed at high temperatures during the infrared sample drying process, because the surface of the graphite oxide contained a large amount of hydroxyl groups and carboxyl groups, the graphite oxide rapidly adsorbed water molecules before and during the infrared test. The absorption peak intensity first increased and then decreased, indicating that the increase in the reaction temperature within a certain range was favorable for the formation of hydroxyl groups and carboxyl groups, and the reaction temperature of 50 °C was favorable for the formation of hydroxyl groups.

**Figure 6:**
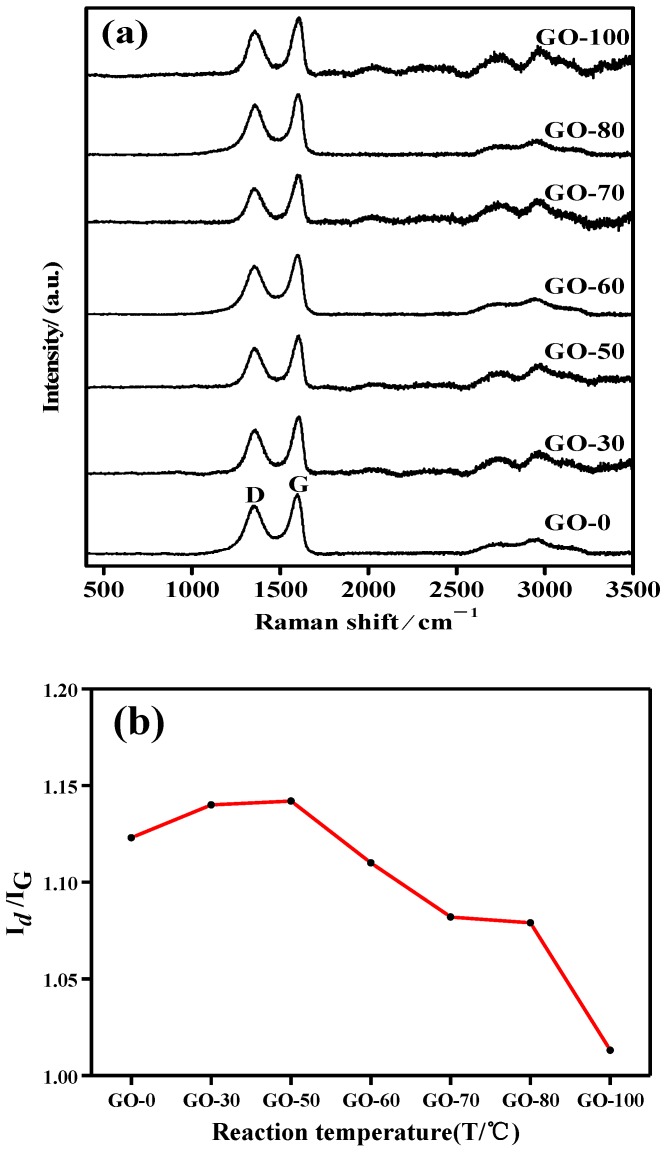
Raman spectrum and D/G peak ratio curve of graphite oxide samples prepared at different reaction temperatures: (**a**) Raman spectrum, (**b**) D/G peak ratio.

The Raman characterizations and D/G peak ratios of graphite oxide samples prepared at different reaction temperatures are shown in Figure 6a,b. As the reaction temperature increased, the I_D_/I_G_ ratio (the intensity ratio of the D peak and the G peak) gradually increased and then rapidly decreased. For the order of the crystal structure of the sample by I_D_/I_G_, the larger the I_D_/I_G_, the more structural defects the sample contains and the lower the degree of ordering [48]. When the reaction temperature was ≤ 50 °C, the graphite could be violently oxidized, and a large number of oxygen-containing functional groups (hydroxyl groups, carboxyl groups, and epoxy groups) were attached to the surface and edge of the graphite, and hydroxyl groups and epoxy groups was dominant. The presence of hydroxyl groups and epoxy groups destroyed the ordered structure of the graphite crystal and the graphite crystal structure defects increased. When the reaction temperature was ≥ 60 °C, the content of hydroxyl groups decreased rapidly due to the changes in the reaction temperature. Although the content of carboxyl groups increased gradually, the increase rate was lower than the decrease rate of hydroxyl groups. The decrease in the functional group promoted the reduction of graphite, thus the graphite crystal structure defects were decreased. When the reaction temperature was 100 °C, because the reaction solution boiled at this time, hydrothermal reduction caused a large amount of oxygen-containing functional groups to be removed, and the graphite crystal structure defects were reduced.

**Figure 7:**
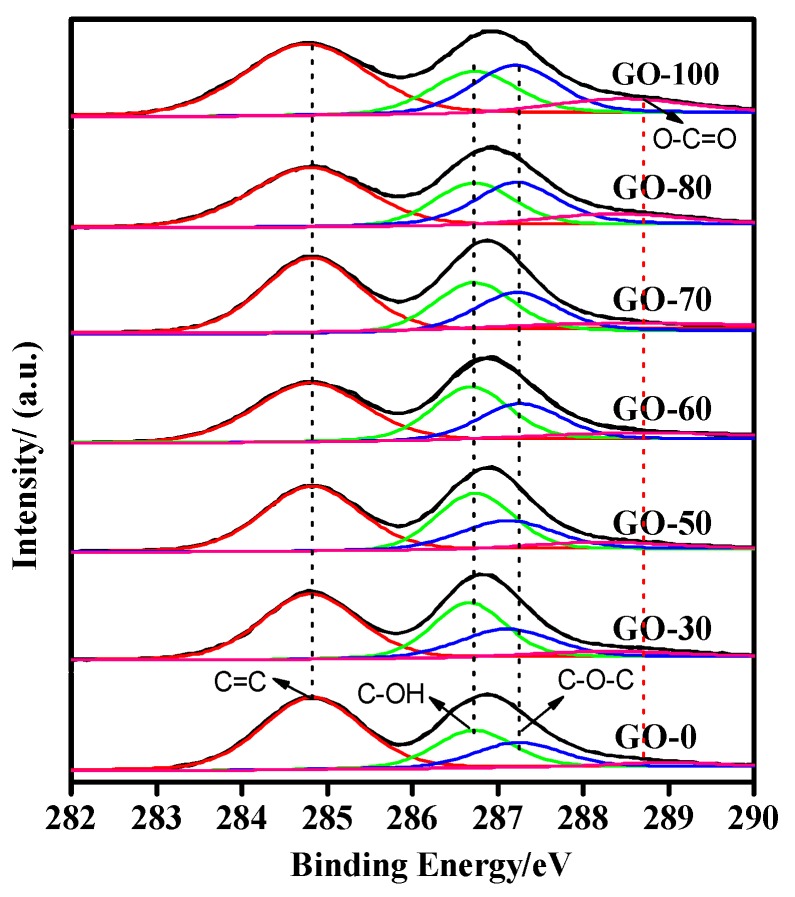
XPS spectra and the content of each functional group of graphite oxide prepared at different reaction temperatures.

The XPS spectra and contents of each functional group of the graphite oxide prepared at different reaction temperatures are shown in Figure 7 and in Table 1. When the reaction temperature was lower, the graphite oxide samples showed a lower degree of oxidation (carbon to oxygen ratio: C/O = 2.09), with less oxygen-containing functional groups on the surface and between layers, as well as low water content and poor hydrophilic ability of samples. Subsequently, as the temperature of the system increased, the degree of oxidation of the graphite oxide samples first increased and then decreased. When the system temperature was 50 °C, the interlayer spacing of the graphite oxide samples was at its highest, the graphite was completely oxidized (C/O = 1.85), and the oxygen-containing functional groups were mainly composed of hydroxyl groups (accounting for approximately 28.88% of the total oxygen-containing functional groups). The high content of hydroxyl and carboxyl groups had good hydrophilic ability and showed the highest moisture content. Excessive temperatures (100 °C) of the system led to the formation of epoxy groups by the decomposition of some hydroxyl groups in the samples, causing the reduction of oxygen-containing functional groups between the graphite layers (100 °C, C/O = 2.23), poor hydrophilic properties, and low moisture content.

From Table 1, we also found that as the reaction temperature increased, the C/O ratio and C=C content first decreased and then increased. This was because as the reaction temperature increased, the C=C content in the graphite decreased, the oxygen-containing functional group content gradually increased, and the oxygen content increased, further causing a decrease in the C/O ratio. Simultaneously, as the reaction temperature increased, the content of C−OH first increased and then decreased. This was because when the reaction temperature was low, C−OH preferentially appeared on the graphite surface, and as the reaction temperature increased, the C−OH content gradually increased. When the reaction temperature was 100 °C, a portion of the C−OH in the graphite oxide was initially removed [49]. Simultaneously, the content of C−O−C and O−C=O increased gradually with the change in the reaction temperature, indicating that the higher the reaction temperature in the high temperature stage, the better the formation of C−O−C and O−C=O. Comparing the changes in the O−C=O and C−OH content, the O−C=O content gradually increased with the increase in the reaction temperature; however, the rate of increase was much smaller than the rate of decrease of C−OH, which further explains that compared to graphite oxide at 50 °C, graphite oxide at 100 °C having a low water content and a large contact angle.

### 3.5. Gas Sensitivity Test

#### 3.5.1. Static Resistance Analysis

**Figure 8:**
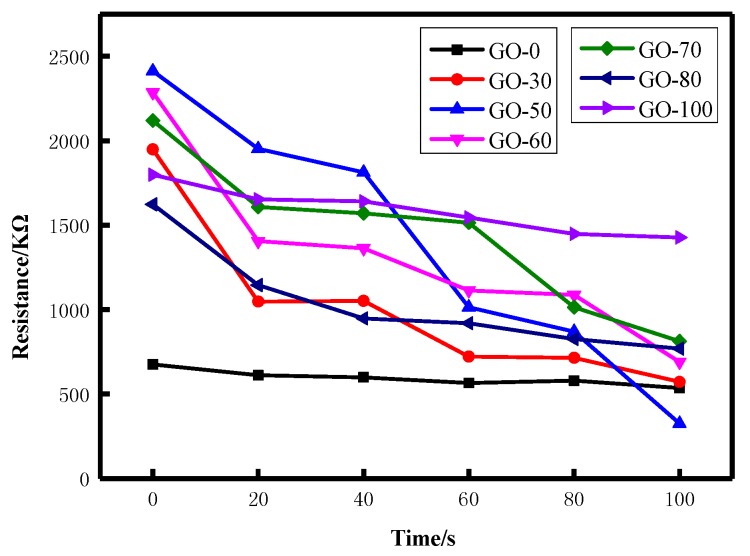
Static resistance curves of graphite oxide samples prepared at different reaction temperatures.

The static resistance changes in the graphite oxide samples prepared at different reaction temperatures at ammonia concentrations of 60 ppm are shown in Figure 8. As can be seen from the figure, as the reaction temperature increased, the initial resistance of the gas sensor first increased and then decreased, and the initial resistance of GO-50 was the largest. This was because the content of oxygen-containing functional groups in the graphite oxide increased first and then decreased with the increase of the reaction temperature. The change of the content of oxygen-containing functional groups caused the change of the degree of defects of the graphite oxide. The fewer the defects, the larger the π electron free path and the better the conductivity. GO-50 has more hydroxyl, carboxyl and epoxy groups and more defects, so it exhibited the largest initial resistance.

When the gas sensor was in ammonia atmosphere at a concentration of 60 ppm, the resistance of the gas sensor decreased, and the resistance of GO-50 sample decreased most significantly. This was because graphite oxide adsorbs water molecules under certain humidity. When ammonia gas contacted GO, it dissolved into the water molecular film to form NH^4+^ and OH^−^, which increased the concentration of conductive ions in the water molecule film and reduced the resistance of the gas sensor. Simultaneously, hydroxyl groups and carboxyl groups of graphite oxide formed a hydrogen bond with the ammonia gas molecule and supplied electrons, resulted in a increase in carrier concentration, a decrease in the band gap width, and an increase in conductivity [50]. The GO-50 sample has a higher content of hydroxyl groups and carboxyl groups. After being placed in the ammonia atmosphere for 100 seconds, the resistance drop of the sample was most pronounced and the resistance was the smallest.

#### 3.5.2. Dynamic Resistance and Sensitivity Analysis

**Figure 9:**
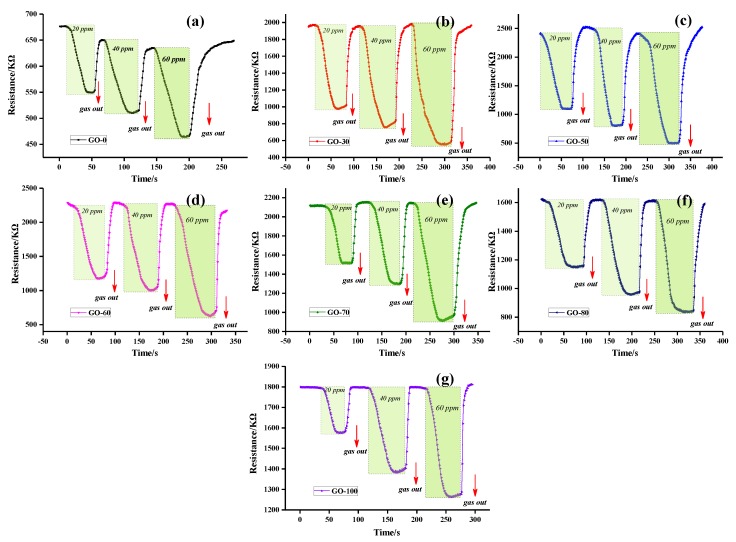
Dynamic resistance curves of graphite oxide samples prepared at different reaction temperatures.

The dynamic continuous response recovery curves of graphite oxide prepared at different reaction temperatures at ammonia concentrations of 20–60 ppm are shown in Figure 9. After adding a certain concentration of ammonia, the resistance of the gas sensor began to decrease sharply, and after a response, the resistance stabilized. The tank was then opened, the gas began to desorb, and the resistance of the gas sensor recovered quickly, which was then restored to the baseline position.

**Figure 10:**
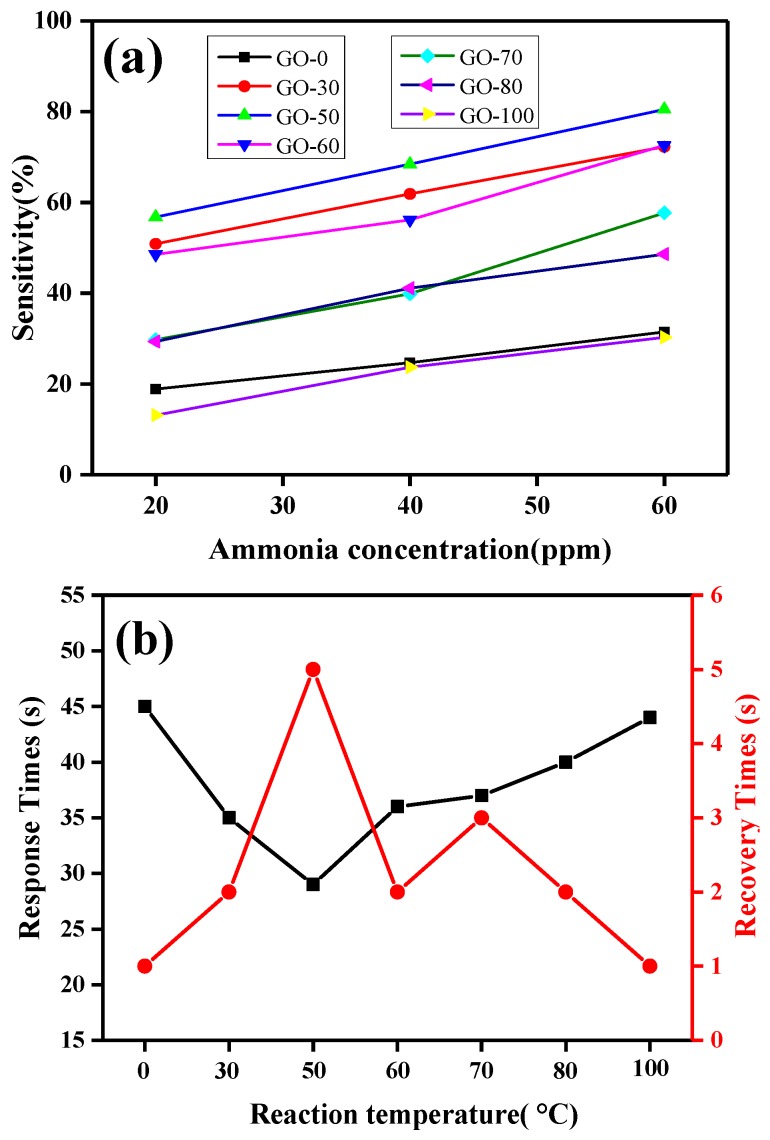
Sensitivity change of graphite oxide and response recovery time curve at 20 ppm ammonia: (**a**) Sensitivity change, (**b**) Response recovery time.

Sensitivity was defined as S = ΔR/R_0_ × 100%, where, R_0_ is the resistance of the gas sensor when it is stable in air, and ΔR is the difference value between the resistance of the gas sensor exposed to a certain concentration of ammonia and R_0_. Figure 10a shows that the sensitivity of all the gas sensors increased linearly with an increase in the concentration of ammonia. When the reaction temperature was 0 °C or ≥70 °C, the sensitivity of the gas sensor (GO-0, GO-70, GO-80, and GO-100) was very low, and the sensitivity of the samples was highest at 60 ppm ammonia concentration, which was 57.72% (GO-70). When the reaction temperature was 30 °C, 50 °C, and 60 °C, the gas sensor showed high ammonia sensitivity. The sensitivity of the GO-50 sample was 56.80% at 20 ppm ammonia concentration, and the sensitivity increased to 80.53% when the ammonia concentration increased to 60 ppm.

The sensitivity of the GO-0 sample was lower than that of the other samples because the reaction temperature was too low, thus the graphite was not completely oxidized, part of the graphite remained, and the contents of the oxygen-containing functional group and the active site were small. The low sensitivity of GO-70, GO-80, and GO-100 samples was due to the decrease in the oxygen content in the samples, resulting in a decrease in the O/C ratio, a decrease in hydroxyl groups content, and a decrease in active sites, which was not conducive to the adsorption of the sample for ammonia. When the reaction temperature was 30 °C, 50 °C, and 60 °C, the sample resistance changed greatly and the sensitivity was high. Thus, the relative content of different oxygen-containing functional groups in graphite oxide plays an important role in influencing sensitivity to ammonia. We found that GO-30, GO-50, and GO-60 had higher hydroxyl groups and carboxyl groups content, with hydroxyl groups and carboxyl groups possessing excellent adsorption characteristics for ammonia [51].

The changes in response time of graphite oxide at 20 ppm ammonia at different reaction temperatures are shown in Figure 10b and Table 2. There was a relationship between response recovery time and sensitivity, i.e., the higher the sensitivity of the sample (e.g., GO-50), the shorter the response time (29 s). In contrast, the lower the sensitivity of the sample (e.g., GO-0), the longer the time (45 s). Simultaneously, the recovery time was inversely related to the response time, i.e., the higher the sensitivity of the component, the longer the recovery time. For example, the GO-50 sample response time was 29 s and the recovery time was the longest (5 s). The analysis showed that the sample with high sensitivity contained relatively more active sites, which can achieve the adsorption equilibrium of ammonia in a short time [50]. Simultaneously, the sensitivity of the sample was influenced by a high content of functional groups and was difficult to desorb after ammonia adsorption.

## 4. Conclusions

The surface of graphite oxide is rich in oxygen-containing functional groups. The present study controlled the preparation conditions of graphite oxide samples to prepare different contents of oxygen-containing functionalized graphite oxide, and a series of graphite oxide gas sensors with different reaction temperatures were prepared by the sol-gel spin-coating process. Changes in the reaction temperatures caused the oxygen-containing functional groups of graphite oxide in the gas sensor to show different trends and exhibit different hydrophilic properties. With the increase of reaction temperature, the C/O ratio and C=C content first decreased and then increased, whereas hydroxyl groups content first increased and then decreased. The higher the temperature, the better formation of the epoxy groups and carboxyl groups, and the reaction temperature of 50 °C was suitable for the preparation of high hydroxyl content graphite oxide. By measuring the ammonia sensitivity of the gas sensor, the change in the content of oxygen-containing functional groups had a significant effect on the conductivity and ammonia sensitivity of the element. The sensitivity of the sample with higher content of hydroxyl groups and carboxyl groups at ammonia concentration of 20 ppm was 56.80% (GO-50), whereas when ammonia concentration was 60 ppm, the sensitivity of the sample increased to 80.53% (GO-50).

## Figures and Tables

**Table 1:** Content of each functional group of graphite oxide prepared at different reaction temperatures.

Sample	Relative Percentage Content /%				C/O
	C=C	C−OH	Epoxy Groups	O−C=O	
GO-0	53.75	23.08	18.03	5.14	2.09
GO-30	43.80	28.77	19.95	7.48	1.90
GO-50	42.58	30.01	19.72	7.69	1.85
GO-60	42.23	27.92	21.33	8.52	1.86
GO-70	44.78	25.27	21.40	8.54	1.89
GO-80	45.42	21.33	22.93	10.31	2.06
GO-100	45.83	20.18	22.96	11.03	2.23

**Table 2:** Response/recovery time, sensitivity value of GO-X to 20 ppm NH_3_.

	GO-0	GO-30	GO-50	GO-60	GO-70	GO-80	GO-100
Response time (s)	45	35	29	36	37	40	44
Recovery time (s)	1	2	5	2	3	2	1
Sensitivity value (%)	18.895	50.867	56.795	48.539	29.742	29.346	13.123

## References

[1] Ohta T., Bostwick A., Mcchesney J., Seyller T., Horn K., Rotenberg E. (2006). Controlling the Electronic Structure of Bilayer Graphene. Science.

[2] Saito R., Fujita M., Dresselhaus G., Dresselhaus M.S. (1992). Electronic structure of chiral graphene tubules. Appl. Phys. Lett..

[3] Mkhoyan K.A., Contryman A.W., Silcox J., Stewart D.A., Eda G., Mattevi C., Miller S., Chhowalla M. (2009). Atomic and Electronic Structure of Graphene-Oxide. Microsc. Microanal..

[4] Li X., Tao L., Chen Z., Fang H., Li X., Wang X., Xu J.B., Zhu H. (2017). Graphene and related two-dimensional materials: Structure-property relationships for electronics and optoelectronics. Appl. Phys. Rev..

[5] Falkovsky L.A. (2008). Optical properties of graphene. J. Exp. Theor. Phys..

[6] Pedersen T.G., Flindt C., Pedersen J., Jauho A.P., Mortensen N.A., Pedersen K. (2009). Optical properties of graphene antidot lattices. Phys. Rev. B Condens. Matter.

[7] Bonaccorso F., Sun Z., Hasan T., Ferrari A.C. (2010). Graphene photonics and optoelectronics. Nat. Photonics.

[8] Zu Y., Tang J., Zhu W., Zhang M., Liu G., Liu Y., Zhang W., Jia M. (2011). Graphite oxide-supported CaO catalysts for transesterification of soybean oil with methanol. Bioresour. Technol..

[9] Gao W. (2015). The Chemistry of Graphene Oxide.

[10] Lobato B., Wendelbo R., Barranco V., Centeno T.A. (2014). Graphite Oxide: An Interesting Candidate for Aqueous Supercapacitors. Electrochim. Acta.

[11] Chen G., Zhai S., Zhai Y., Zhang K., Yue Q., Wang L., Zhao J., Wang H., Liu J., Jia J. (2011). Preparation of sulfonic-functionalized graphene oxide as ion-exchange material and its application into electrochemiluminescence analysis. Biosens. Bioelectron..

[12] Hontoria-Lucas C., López-Peinado A.J., López-González J.D.D., Rojas-Cervantes M.L., Martín-Aranda R.M. (1995). Study of oxygen-containing groups in a series of graphite oxides: Physical and chemical characterization. Carbon.

[13] Huang Q., Sun H.J., Yang Y.H. (2011). Spectroscopy Characterization and Analysis of Graphite Oxide. Chin. J. Inorg. Chem..

[14] Zhao X., Johnson J.K. (2005). An Effective Potential for Adsorption of Polar Molecules on Graphite. Mol. Simul..

[15] Zhang B., Li F., Wu T., Sun D., Li Y. (2015). Adsorption of p-nitrophenol from aqueous solutions using nanographite oxide. Colloids Surf. A Physicochem. Eng. Asp..

[16] Hsiao M.C., Liao S.H., Yen M.Y., Liu P.I., Pu N.W., Wang C.A., Ma C.C. (2010). Preparation of covalently functionalized graphene using residual oxygen-containing functional groups. ACS Appl. Mater. Interfaces.

[17] Chen Y., Zhang Z., Huang Z., Zhang H. (2016). Effects of oxygen-containing functional groups on the supercapacitor performance of incompletely reduced graphene oxides. Int. J. Hydrogen Energy.

[18] Wang S., Cole I.S., Zhao D., Li Q. (2016). The dual roles of functional groups in the photoluminescence of graphene quantum dots. Nanoscale.

[19] Staudenmaier L., Dtsch B. (1898). The structure of graphite oxide: Investigation of its surface chemical groups. Chem. Ges.

[20] Nakajima T., Matsuo Y. (1994). Formation process and structure of graphite oxide. Carbon.

[21] Nakajima T., Mabuchi A., Hagiwara R. (1988). A new structure model of graphite oxide. Carbon.

[22] Beckett R.J., Croft R.C. (1952). The Structure of Graphite Oxide. J. Phys. Chem..

[23] Mermoux M., Chabre Y., Rousseau A. (1991). FTIR and 13 C NMR study of graphite oxide. Carbon.

[24] Avdeev V.V., Sorokina N.E., Nikol’ Skaya I.V., Monyakina L.A., Voronkina A.V. (1997). Synthesis of Intercalation Compounds in the System Graphite-HNO_3_-H_2_SO_4_. Inorg. Mater..

[25] Vieira M.A., Gonçalves G.R., Cipriano D.F., Schettino M.A., Filho E.A.S., Cunha A.G., Emmerich F.G., Freitas J.C.C. (2016). Synthesis of graphite oxide from milled graphite studied by solid-state 13 C nuclear magnetic resonance. Carbon.

[26] Hamwi A., Marchand V. (1996). Some chemical and electrochemical properties of graphite oxide. J. Phys. Chem. Solids.

[27] Jeong H.K., Lee Y.P., Lahaye R.J., Park M.H., An K.H., Kim I.J., Yang C.W., Park C.Y., Ruoff R.S., Lee Y.H. (2008). Evidence of Graphitic AB Stacking Order of Graphite Oxides. J. Am. Chem. Soc..

[28] Fan X., Peng W., Li Y., Li X., Wang S., Zhang G., Zhang F. (2008). Deoxygenation of Exfoliated Graphite Oxide under Alkaline Conditions: A Green Route to Graphene Preparation. Adv. Mater..

[29] Boehm H.P., Clauss A., Hofmann U. (1961). Graphite oxide and its membrane properties. J. Chim. Phys..

[30] He H., Riedl T., Lerf A., Klinowski J. (1996). Solid-State NMR Studies of the Structure of Graphite Oxide. J. Phys. Chem..

[31] He H., Klinowski J., Forster M., Lerf A. (1998). A new structural model for graphite oxide. Chem. Phys. Lett..

[32] Lerf A., He H., Forster M., Klinowski J. (1998). Structure of Graphite Oxide Revisited. J. Phys. Chem. B.

[33] Lerf A., Buchsteiner A., Pieper J., Schöttl S., Dekany I., Szabo T., Boehm H.P. (2006). Hydration behavior and dynamics of water molecules in graphite oxide. J. Phys. Chem. Solids.

[34] Dékány I., Krüger-Grasser R., Weiss A. (1998). Selective liquid sorption properties of hydrophobized graphite oxide nanostructures. Colloid Polym. Sci..

[35] Ramesh P., Bhagyalakshmi S., Sampath S. (2004). Preparation and physicochemical and electrochemical characterization of exfoliated graphite oxide. J. Colloid Interface Sci..

[36] Peckett J.W., Trens P., Gougeon R.D., Pöppl A., Harris R.K., Hudson M.J. (2000). Electrochemically oxidised graphite: Characterisation and some ion exchange properties. Carbon.

[37] De Wit M., Vanneste E., Blockhuys F., Verreyt G., Tachelet W., Nagels L.J., Geise H.J. (2000). Chemically Sensitive Sensor Comprising Arylene Alkenylene Oligomers. U.S. Patent.

[38] Drewniak S., Muzyka R., Stolarczyk A., Pustelny T., Kotyczka-Moraå Ska M., Setkiewicz M. (2016). Studies of Reduced Graphene Oxide and Graphite Oxide in the Aspect of Their Possible Application in Gas Sensors. Sensors.

[39] Wang J., Kwak Y., Lee I.Y., Maeng S., Kim G.H. (2012). Highly responsive hydrogen gas sensing by partially reduced graphite oxide thin films at room temperature. Carbon.

[40] Yeh T.F., Syu J.M., Cheng C., Chang T.H., Teng H. (2010). Graphite Oxide as a Photocatalyst for Hydrogen Production from Water. Adv. Funct. Mater..

[41] Xu C., Cao Y., Kumar R., Wu X., Wang X., Scott K. (2011). A polybenzimidazole/sulfonated graphite oxide composite membrane for high temperature polymer electrolyte membrane fuel cells. J. Mater. Chem..

[42] Travlou N.A., Kyzas G.Z., Lazaridis N.K., Deliyanni E.A. (2013). Graphite oxide/chitosan composite for reactive dye removal. Chem. Eng. J..

[43] Zhao Y., Tang G.S., Yu Z.Z., Qi J.S. (2012). The effect of graphite oxide on the thermoelectric properties of polyaniline. Carbon.

[44] Wang Q., Sun H., Peng T. (2016). Structure Development during the Cation Exchange Processes of Graphite Oxide. Acta Phys. -Chim..

[45] Wang P., Sun H., Peng T. (2015). The Evolution Rule of Three-Dimensional Structures of Graphite During Oxidation. Nano Brief Rep. Rev..

[46] Sun H., Peng T. (2015). The Preparation of Graphene Materials via Oxidation-Reduction.

[47] Cotton F.A., Wilkinson G. (1988). Advance Inorganic Chemistry. J. Chem. Educ..

[48] Ferrari A.C., Robertson J. (2000). Interpretation of Raman spectra of disordered and amorphous carbon. Phys. Rev. B.

[49] Ding J.N., Liu Y.B., Yuan N.Y., Ding G.Q., Fan Y., Yu C.T. (2012). The influence of temperature, time and concentration on the dispersion of reduced graphene oxide prepared by hydrothermal reduction. Diam. Relat. Mater..

[50] Lu G., Ocola L.E., Chen J. (2009). Reduced graphene oxide for room-temperature gas sensors. Nanotechnology.

[51] Yue P., Li J. (2013). Ammonia adsorption on graphene and graphene oxide: A first-principles study. Front. Environ. Sci. Eng..

